# The Rise of Inflow Cisternostomy in Resource-Limited Settings: Rationale, Limitations, and Future Challenges

**DOI:** 10.1155/2021/6630050

**Published:** 2021-01-08

**Authors:** Ulrick Sidney Kanmounye

**Affiliations:** Department of Research, Association of Future African Neurosurgeons, Yaounde, Cameroon

## Abstract

Low- and middle-income countries (LMICs) bear most of the global burden of traumatic brain injury (TBI), but they lack the resources to address this public health crisis. For TBI guidelines and innovations to be effective, they must consider the context in LMICs; keeping this in mind, this article will focus on the history, pathophysiology, practice, evidence, and implications of cisternostomy. In this narrative review, the author discusses the history, pathophysiology, practice, evidence, and implications of cisternostomy. Cisternostomy for the management of TBI is an innovation developed in LMICs, primarily for LMICs. Its practice is based on the cerebrospinal fluid shift edema theory that attributes injury to increased pressure within the subarachnoid space due to subarachnoid hemorrhage and subsequent dysfunction of glymphatic drainage. Early reports of the technique report significant improvements in the Glasgow Outcome Scale, lower mortality rates, and shorter intensive care unit durations. Most reports are single-center studies with small sample sizes, and the technique requires experience and skill. These limitations have led to criticisms and slow adoption of the technique. Further research is needed to establish the effect of cisternostomy on TBI outcomes.

## 1. Introduction

The burden of traumatic brain injury (TBI) is enormous and disproportionate. TBI causes 111 years of life lived with disability per 100 000, and 80% of its burden occurs in low- and middle-income countries (LMICs) [1]. In LMICs, the burden of TBI, and those classified as severe, is aggravated by lack of resources for efficient prevention and management [2].

Moreover, there is a disparity in TBI research and innovation. Most TBI research, guidelines, and innovations are developed in high-income countries (HICs), where TBI management's epidemiology and resources are more favorable [3–5]. Consequently, international TBI guidelines are either inconsistently implemented or not implemented in most LMICs [6–9]. To curtail TBI's burden in LMICs, stakeholders must develop and implement holistic health systems strengthening policies, and evidence-based TBI guidelines should equally be mindful of the local context [10].

Cisternostomy is one of the few innovations in the field of neurotraumatology developed in LMICs for use in resource-limited settings. Keeping this in mind, this article will describe the history, rationale, technique, indications, and efficacy of cisternostomy for severe TBI.

## 2. History

Cisternostomy in the context of severe TBI aims at opening the basal cisterns to atmospheric pressure and tackle the vicious process leading to posttraumatic brain swelling [11]. There are two types of cisternostomy based on the mechanism of action: outflow (ventriculocisternostomy and cystocisternostomy) and inflow (cisternostomy proper) [12]. Outflow cisternostomies were the first to be described in modern neurosurgery. Arne Torkildsen performed the first successful ventriculocisternostomy in 1937 for cerebrospinal fluid (CSF) diversion, and the intervention was the preferred treatment of noncommunicating hydrocephalus after World War II [13, 14]. The idea of inflow cisternostomy was developed in the context of vascular neurosurgery and still represents a valuable microsurgical step routinely carried out during clipping of anterior circulation aneurysms [15]. The first mention of inflow cisternostomy for the management of severe TBI was in 2012 by Dr. Cherian from Nepal [16].

## 3. Rationale

The idea of offering cisternostomy to TBI patients is intertwined with the discovery of the glymphatic system. The glymphatic system is a network of perivascular channels that promote entry and exit of substances within the CNS [17]. The fluid in the glymphatic system is made from CSF produced by the choroid plexus and circulates within the subarachnoid space into perivascular (Virchow–Robin) spaces. The interstitial fluid collected within perivenous spaces is then drained to the cervical lymphatic circulation [18]. TBI affects the flow and composition of extracellular fluids within the central nervous system (CNS). It damages the glymphatic system and causes biomarkers' liberation (glial fibrillary acidic protein, neuron-specific enolase, and S100 calcium-binding protein B) and waste CNS products [19]. Acute TBI causes the translocation of type 4 aquaporin channels away from astrocytes' endfeet, thereby altering the flow of extracellular CNS fluid (glymphatic and interstitial) and causing reactive astrogliosis [20].

Based on these facts, the concept of CSF shift edema started to emerge, as it was suggested that, following posttraumatic subarachnoid bleeding which impairs the normal CSF flow and resorption, the interstitial and intracellular fluid could increase as a result of the shift from the rising pressure of the basal cisterns into the brain parenchyma [11]. A similar concept has been described in acute ischemic stroke edema. Mestre et al. [21] tracked CSF flow in mice after middle cerebral artery stroke and found evidence of CSF shift edema in the ipsilateral hemisphere. Therefore, the rationale of cisternostomy is to open and rinse the basal cisterns allowing a removal of blood products and addressing the altered gradient pressure between subarachnoid space and the brain parenchyma [11].

Numerous authors have studied the association between subarachnoid CSF flow and TBI. For example, the role of cisternotomy on glymphatic flow and TBI was studied in mice models by Plog et al. [22] who used horizontal cisternotomy to drain CSF from mice that had acute TBI continually. Of note, they found no evidence in favor of cisternostomy preventing the secondary cascade of TBI. The reason is that CSF drainage by the cisterna magna cisternotomy reduces the hydraulic pressure that drives fluid exchange between CSF and interstitial fluid [17]. As a result, it inhibits glymphatic efflux, which alters TBI biomarkers' clearance and waste products. Unlike cisterna magna cisternotomy, cisternostomy exposes more cerebral cisterns (interoptic, optico-carotid, lateral carotid, interpeduncular, and prepontine) to atmospheric pressure and removes blood products from the subarachnoid space. Traumatic subarachnoid hemorrhage occurs in 11–60% of TBI cases due to injury to subarachnoid vessels [23, 24]. This might explain the efficacy of cisternostomy in some TBI cases. Another important consideration is the timing of TBI-related glymphatic dysfunction. Glymphatic disruption occurs between days 3 and 28 in mice models, although in a small number of cases, it occurs as early as day 1 [19]. Therefore, if cisternostomy could in the future prove its effectiveness in all TBI cases, some of its effects could not be simply explained by the CSF shift edema theory alone and should perhaps be attributable to the reduced intracranial pressure and the overall optimization of the CSF flow as seen following decompressive craniectomy [25].

## 4. Practice of Cisternostomy for TBI Management

Cisternostomy is always performed in conjunction with DC, and such approach represents the last resource in the treatment of medically refractory severe TBI [3, 26, 27].

In the seminal report on the advantages of cisternostomy, Cherian et al. [28] reported lower mortality (15.6% in the cisternostomy group vs. 26.4% in the DC and cisternostomy group vs. 34.8% in the DC group), shorter mechanical ventilation times (2.4 days in the cisternostomy group vs. 3.2 days in the DC and cisternostomy group vs. 6.3 days in the DC group), and better Glasgow outcome scales at 6 weeks (3.9 in the cisternostomy group vs. 3.7 in the DC and cisternostomy group vs. 2.8 in DC group) [28]. Unsurprisingly, the adoption of cisternostomy has increased significantly in the past 8 years in several LMICs, diffusing from its birthplace, Nepal, to neurosurgical units in Brazil, China, Egypt, India, Iran, and Iraq [28–35].

Shorter mechanical ventilation and intensive care unit times are most needed in resource-limited settings where most TBI cases tend to happen [30, 36]. Cisternostomy poses a lesser risk and cost than DC because DC must be followed by a second intervention, a cranioplasty, which carries its own risk and cost [30, 37]. However, we note that economic comparisons have been limited to direct expenses, and no study has compared the cost-effectiveness of cisternostomy and DC. A cost-effectiveness analysis will factor in TBI's societal cost and benefit (mortality and morbidity averted by both interventions) and ascertain the economic superiority of one intervention over the other [38–43]. Another consideration is technicality and resource availability. Cisternostomy is a complex microsurgical procedure, and few LMIC neurosurgeons have the experience and equipment to perform cisternostomy safely [32, 33, 44, 45]. The expansion of cisternostomy in LMICs will require capacity building and increased access to microscopes in the form of fellowships and the development of low-cost microscopes [38–43].

## 5. Quality of Evidence

Most published cisternostomy studies are either observational (retrospective and nonrandomized), run in single centers, or have small sample sizes [29–35]. This diminishes the quality of evidence generated and has precluded their inclusion in TBI meta-analyses and guidelines [3, 4]. Further studies are needed for cisternostomy to be accepted as an option for TBI management. Future cisternostomy must be robust, i.e., ideally, they must be randomized multicentric studies with low risk of bias in randomization and concealment and a large sample size (i.e., ≥100 patients) [46]. Although randomized control trials are the preferred study design for quality evidence, they are not always feasible. Randomized control trials can be impractical, costly, and lengthy [47]. For these reasons, a significant proportion of evidence in neurosurgery spawns from cohort, case-control, and quasi-experimental studies [47]. The evidence from these studies can be valuable if they are designed to minimize bias [47–50].

## 6. Implications and Future Direction

Cisternostomy is the epitome of TBI innovation for LMICs and by LMICs. The inventor of this technique is an LMIC neurosurgeon, and most publications on the topic are from LMICs. This experience lays the ground for LMIC research collaborations in the form of large multicentric (randomized, cohort, case-control, or quasi-experimental) studies. The evidence generated from robust studies will facilitate a more widely and consistent adoption of cisternostomy and will eventually improve the technique by generating new research questions, such as the usefulness of relying on intraoperative ultrasound to visualize the cisterns in a swollen brain. [51].

## 7. Conclusions

The management of TBI is complex, even more so in resource-limited settings. To solve the public health and clinical problem posed by TBI, LMIC researchers must be ready to innovate. The use of cisternostomy in the surgical management of severe TBI represents a revolutionary step, and disruptive theory of CSF shift edema has already contributed lessons to the entire neurotrauma community. LMIC TBI researchers and innovators can build on the cisternostomy experience to develop context-specific and evidence-based solutions.

## Data Availability

No data were used to support this study.
